# Prognostic histologic subtyping of dominant tumor in resected synchronous multiple adenocarcinomas of lung

**DOI:** 10.1038/s41598-021-88193-9

**Published:** 2021-05-05

**Authors:** Ping-Chung Tsai, Chia Liu, Yi-Chen Yeh, Chun-Ku Chen, Po-Kuei Hsu, Hui-Shan Chen, Chien-Sheng Huang, Chih-Cheng Hsieh, Han-Shui Hsu, Biing-Shiun Huang

**Affiliations:** 1 Division of Thoracic Surgery, Department of Surgery, Taipei Veterans General Hospital, Taipei, Taiwan; 2 Department of Pathology, Taipei Veterans General Hospital, Taipei, Taiwan; 3 Department of Radiology, Taipei Veterans General Hospital, Taipei, Taiwan; 4 Department of Health Care Administration, Chang Jung Christian University, Tainan, Taiwan; 5 School of Medicine, National Yang-Ming Chiao Tung University, Hsinchu, Taiwan; 6 Institute of Clinical Medicine, School of Medicine, National Yang-Ming University, 201, Section 2, Shih-Pai Road, Taipei, Taiwan

**Keywords:** Oncology, Cancer, Cancer therapy, Lung cancer

## Abstract

The prognostic role of histological patterns of dominant tumor (DT) and second dominant tumor (sDT) in synchronous multiple adenocarcinoma (SMADC) of lung remains unclear. SMADC patients diagnosed between 2003 and 2015 were retrospectively reviewed. DT and sDT were defined as two maximum diameters of consolidation among multiple tumors. Histological pattern was determined using IASLC/ATS/ERS classification system. DTs were divided into low- (lepidic), intermediate- (acinar, papillary) and high-grade (micropapillary, solid) subtypes, and sDTs into non-invasive predominant (lepidic) and invasive predominant (acinar, papillary, micropapillary, solid) subtypes. During mean 74-month follow-up among 149 nodal-negative patients having SMADC resected, recurrence was noted in 44 (29.5%), with significantly higher percentage in high-grade DT (*p* < 0.001). Five-year overall (OS) and disease-free (DFS) survivals in low-, intermediate- and high-grade DT were 96.9%, 94.3%, 63.3% (*p* < 0.001) and 100%, 87.2%, 30.0%, respectively (*p* < 0.001). Cox-regression multivariate analysis demonstrated high-grade DT as a significant predictor for DFS (Hazard ratio [HR] 5.324; 95% CI 2.570–11.462, *p* < 0.001) and OS (HR 3.287; 95% CI 1.323–8.168, *p* = 0.010). Analyzing DT and sDT together, we found no significant differences in DFS, either in intermediate- or high-grade DT plus invasive or non-invasive sDT. DT was histologically an independent risk factor of DFS and OS in completely resected nodal-negative SMADCs.

## Introduction

The incidence of synchronous multiple primary lung cancer (SMPLC) in patients with non-small cell lung cancers varies from 0.2 to 20%^1^, and tends to increase with the use of higher-resolution precise chest imaging techniques, especially in those lesions containing ground glass opacities (GGOs) strongly indicative of adenocarcinomas of lung. Modified criteria of Martini and Melamed have been used to define SMPLC based on different histology or location of different lobes without lymphatic or systemic metastasis^2,3^, comprehensive histologic subtyping^4^, radiological component of GGO^5^, or even genomic heterogeneity^6^. Surgical outcomes for SMPLCs have been reported as being acceptable and compatible for patients with solitary primary lung cancer^4,7^. While surgical treatment planning is crucial in SMPLC approach, additional studies have documented that the dominant tumor (DT) in SMPLCs plays a critical prognostic role and should be determined based on the solid components on high-resolution computed-tomography and fluorodeoxyglucose uptake by positron emission tomography^8^.

For solitary lung adenocarcinoma, the International Association for the Study of Lung Cancer/American Thoracic Society/European Respiratory Society (IASLC/ATS/ERS) classification based on histologic subtypes has proved its significant prognostic and predictive value^3^. Accordingly, patients with high-grade subtypes presenting as micropapillary or solid predominant pattern have a significantly higher risk for recurrence in resected stage I and death in all stages of lung adenocarcinoma^9^. Currently, evaluation of the prognostic role of DT and second dominant tumor (sDT) in SMPLCs has focused especially on the radiologic features^10,11^. However, the prognostic role of the IASLC/ATS/RES histological classification of synchronous multiple adenocarcinoma (SMADCs) remains unclear. This study aimed to characterize the prognostic impact of DT plus sDT tumor pattern as histologic characteristics in SMADCs.

## Patients and methods

### Study design and patient selection

Patients with pathologically proven multiple primary lung cancer treated at Taipei Veterans General Hospital from May 2003 to December 2015 were included. Patient data were obtained from medical records and were analyzed retrospectively. Demographic and clinical characteristics included age, sex, smoking history, preoperative serum carcinoembryonic antigen (CEA) level (normal range: < 6 ng/mL), histologic tumor type, tumor size, tumor location, tumor differentiation, radiologic features of the DT (pure GGO, GGO-dominant, solid-dominant), presence of lympho-vascular invasion, presence of pleural invasion, and whether adjuvant chemotherapy was administrated. The study protocol and informed consent waiver were approved by the Institutional Review Board of Taipei Veterans General Hospital (Approval No. 2020-05-001CC). All research was performed in accordance with relevant guidelines and regulations.

**Figure 1:**
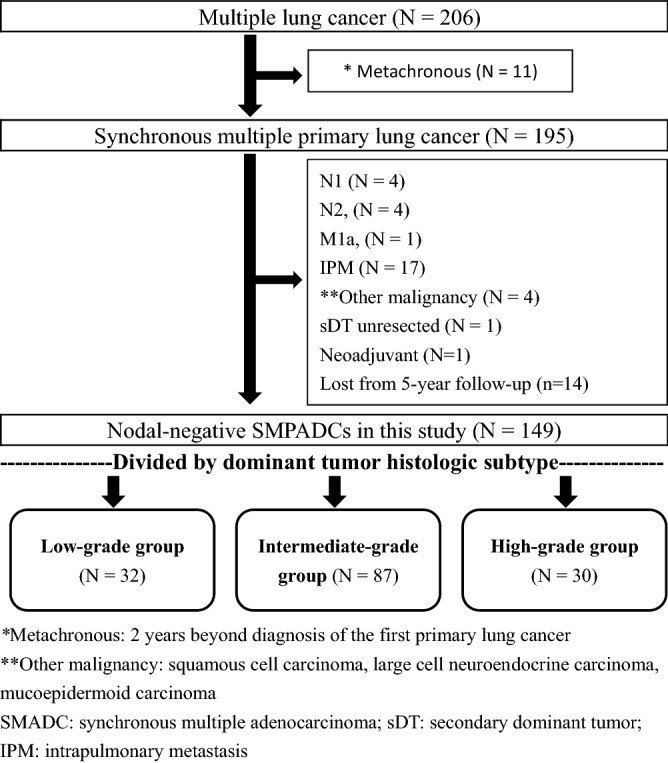
Flow diagram of the selection of patients with synchronous multiple primary adenocarcinomas (SMADCs) included in the present study.

During the study period, 206 patients who met the inclusion criteria of having pathologically proven multiple primary lung cancer were extracted from the hospital’s prospective registered database. Patients in any of the following cases were excluded from the study: (i) a second tumor was absent on the original CT scan, or diagnosed 2 years beyond the diagnosis of the first primary lung cancer, as metachronous lung cancer did; (ii) patients were nodal-positive or had distant metastasis after DT resection; (iii) patients had histologic pathology other than lung adenocarcinoma, secondary tumor without surgical resection, intrapulmonary metastasis (IPM) or received neoadjuvant chemotherapy; and (iv) patients were not routinely followed-up in the first 5 years. Finally, 149 patients with SMADCs were included for further analysis (Fig. 1). Among them, 109 had their tumors resected in an episode of operation (two patients were with bilateral tumors) and 40 had their tumors resected at intervals from 1 to 23 months (36 patients were with bilateral tumors).

### Preoperative radiologic evaluation

Radiologic evaluation was conducted by thin-section CT or involved multidimensional slicing and reconstruction into axial, coronal, and sagittal views. Tumor characteristics from the preoperative chest CT were read by two independent observers and tumor size was reviewed in detail. The extent of GGO was also evaluated in all tumors using the same thin-section CT scan with a 2 mm collimation (GE Healthcare, Chicago, IL, USA). The lung was photographed with a window level of − 500 to – 700 H and a window depth of 1000 to 2000 H as the “lung window,” and a window level of 30 to 60 H and a window depth of 350 to 600 H as the “mediastinal window.” Consolidation tumor ratio (CTR) was defined as the ratio of the maximum size of consolidation to the maximum tumor size on thin-section CT scan. Tumors were then classed into three groups: pure GGO (CTR = 0, no focal nodular opacity), GGO-dominant (0 < CTR ≤ 0.5) and solid-dominant (0.5 < CTR ≤ 1.0), for survival analysis taking CTR as an independent variable. DT and sDT were determined according to the maximum diameter of consolidation on the lung window among multiple primary lung cancers.

### Pathology histology

Pathologic stage was determined using the 8th edition of the AJCC TNM classification system for lung cancer. Histological subtyping of specimens was made by review of archived hematoxylin and eosin stain slides by experienced pathologists according to the 2015 WHO lung adenocarcinoma classification system. The predominant histological subtypes in each category were determined as the ones with the highest percentage of component. DTs showing purely lepidic (adenocarcinoma in situ), mostly lepidic (minimally invasive adenocarcinoma) or predominantly lepidic patterns were defined as low-grade group. Acinar and papillary patterns were defined as intermediate-grade group. Micropapillary and solid patterns were defined as high-grade group. In sDTs, predominant patterns were divided into non-invasive group (lepidic) and invasive group that was non-lepidic predominant (acinar, papillary, micropapillary, solid).

### Patient follow-up

Postoperative surveillance was scheduled every 3 months for the first 2 years, every 6 months for the third to fifth year, and annually thereafter. Chest CT was performed every 6 months for 5 years, then annually. Locoregional recurrence was defined as tumor recurrence on a contiguous anatomic site, including the ipsilateral hemithorax and mediastinum, after surgical resection. Distant recurrence was defined as a tumor recurring in the contralateral lung or outside the hemithorax and mediastinum after surgical resection. Recurrences were confirmed by tissue biopsy or clinically determined by the multidisciplinary lung cancer committee. For patients highly suspected of having local or distal metastasis that developed after operation, CT-guided or surgical biopsy was performed for tissue diagnosis if indicated, and comprehensive histology was compared with the original tumor to distinguish it from metastatic tumor tissue.

### Statistical analysis

Differences between categorical variables were assessed using χ2 test and Fisher’s exact test. For discrete and continuous variables, unpaired Student’s t-test was used. Univariable and multivariable cox-regression analyses were used to identify predictors of pathologic results. A *p* value less than 0.05 was established as statistical significance. All statistical analyses were performed using SPSS 25.0 statistical software (IBM Corp, Armonk, NY, USA).

## Results

The baseline demographic and clinical characteristics of the study population are described in Table 1. All patients underwent resection of at least 2 tumors and were diagnosed pathologically as having multiple primary lung adenocarcinoma. Among these 149 patients, 32 (21.5%) were classified according to their DT histologic subtype into “low-grade” group, 87 (58.4%) were classified into “intermediate-grade” group and 30 (20.1%) were classified into “high-grade” group. Meanwhile, 32 (100%), 43(49.4%) and 6 (20.0%) sDTs in the low-, intermediate- and high-grade groups were classified into non-invasion group, respectively. The histologic relevance between DTs and sDTs is illustrated in Supplementary Fig. 1. The association between the characteristics of OS or DFS and IASLC/ATS/ERS classification of the DT group is summarized in Table 2. Mean follow-up in all patients was 80.4 ± 34.8 months (median: 74 months) and disease-free interval was 69.4 ± 37.1 months. The high-grade DT group had the poorest 5-year OS (63.3%, *p* < 0.001), 5-year DFS (30.0%, *p* < 0.001), and total recurrence rate (n = 24, 80.0%, *p* < 0.001). Compared with the intermediate group, the high-grade DT group showed no significant difference in patterns of recurrence, local or distant metastasis.

**Table 1:** Demographic and clinical characteristics of 149 patients with SMADCs, grouped by IASLC/ATS/ERS classification of dominant tumor (DT).

Group variables		Low-grade (lepidic; %)	Intermediate-grade (acinar, papillary; %)	High-grade (micropapillary, solid;%)	*p* value
**Number**	149 (100%)	32 (21.5)	87 (58.4)	30 (20.1)	
**Age (years old)**	62.0 ± 10.8	58.5 ± 9.6	62.1 ± 11.1	65.6 ± 10.3	0.033
**Gender**					0.112
Male	54	7 (21.9)	33 (37.9)	14 (46.7)	
Female	95	25 (78.1)	54 (62.1)	16 (53.3)	
**Smoking status (yes)**					0.018
Previously or currently	41	5 (15.6)	22 (25.3)	14 (46.7)	
Non-smoker	108	27 (84.4)	65 (74.7)	16 (53.3)	
**Preoperative CEA level (ng/mL)**					0.030
≥ 6.0 ng/mL	22	3 (9.4)	10 (11.5)	9 (30.0)	
< 6.0	127	29 (90.6)	77 (88.5)	21 (70.0)	
**Tumor size (cm)**	2.26 ± 1.34	1.29 ± 0.69	2.35 ± 1.27	3.01 ± 1.47	< 0.001
**Resected tumor number(s)**	2.4 ± 1.0(2–9)	2.8 ± 1.2	2.6 ± 1.5	2.4 ± 1.0	0.436
**Radiologic appearance of DT**					< 0.001
Pure GGO	34	21 (65.6)	13 (14.9)	0	
GGO-dominant	39	10 (31.3)	26 (29.9)	3 (10.0)	
Solid-dominant	76	1 (3.1)	48 (55.2)	27 (90.0)	
**Laterality**					0.172
Ipsilateral	111	27 (84.4)	60 (69.0)	24 (80.0)	
Bilateral	38	5 (15.6)	27 (31.0)	6 (20.0)	
**Tumor location**					0.800
At different lobe	100	20 (62.5)	60 (69.0)	20 (66.7)	
At the same lobe	49	12 (37.5)	27 (31.0)	10 (33.3)	
**TNM stage (AJCC 8th)**					< 0.001
pTis	12	12	0	0	
pT1a	15	5	9	1	
pT1b	21	3	18	0	
pT1c	14	4	9	1	
pT2a	74	8	45	21	
pT2b	8	0	5	3	
pT3	4	0	0	4	
pT4	1	0	1	0	
**Pleural invasion**					< 0.001
P0	71	24 (75.0)	41 (47.1)	6 (20.0)	
P1 + P2 + P3	78	8 (25.0)	46 (52.9)	24 (80.0)	
**Differentiation**					< 0.001
Well + moderate	97	31 (96.9)	61 (70.1)	5 (16.7)	
Poorly	52	1 (3.1)	26 (29.9)	25 (83.3)	
**Lymphovascular invasion**					< 0.001
Nil	119	32 (100)	72 (82.8)	15 (50.0)	
Yes	30	0	15 (17.2)	15 (50.0)	
**Adjuvant chemotherapy**					< 0.001
Nil	96	31 (96.9)	55 (63.2)	10 (33.3)	
Yes	53	1 (3.1)	32 (36.8)	20 (66.7)	
**2nd dominant tumor**					< 0.001
Non-invasive (lepidic)	81	32 (100)	43 (49.4)	6 (20.0)	
Invasive (non-lepidic)	68	0	44 (50.6)	24 (80.0)	

*DT* dominant tumor of SMADCs, *AIS* adenocarcinoma in situ, *MIA* minimal invasive adenocarcinoma, *CEA* carcinoembryonic agent.

**Table 2:** Association between survival and IASLC/ATS/ERS classification of dominant tumor (DT).

Variables	Grade (N = 149); %	Low (N = 32); %	Intermediate (N = 87); %	High (N = 30); %	*p* value
5-year overall survival (%)	88.6	96.9	94.3	63.3	< 0.001
5-year disease-free survival (%)	78.5	100	87.2	30.0	< 0.001
Follow-up period (months)	80.4 ± 34.8	66.3 ± 23.2	89.5 ± 36.8	69.0 ± 31.6	0.001
Disease-free duration (months)	69.4 ± 37.1	66.3 ± 23.2	80.9 ± 37.9	39.6 ± 29.5	< 0.001*
Total recurrence, no.	44 (29.5)	0	20 (23.3)	24 (80.0)	< 0.001
Patterns of recurrence					0. 595**
Local only	13 (29.5)	0	7 (35.0)	6 (25.0)	
Distant only	12 (27.3)	0	6 (30.0)	6 (25.0)	
Local + Distant	19 (43.2)	0	7 (35.0)	12 (50.0)	

*ANOVA Test.

**Chi-Square tests, compared between intermittent and high grade groups.

**Figure 2:**
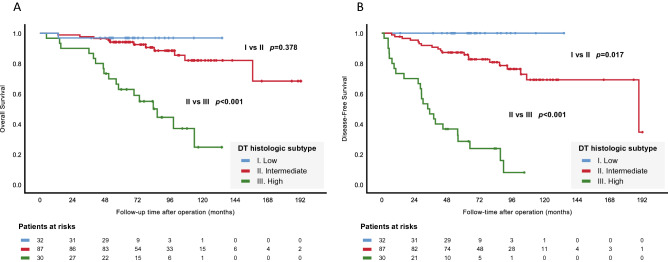
Overall and disease-free survival between different histologic subtypes (**A**, **B**) of the dominate tumor.

**Figure 3:**
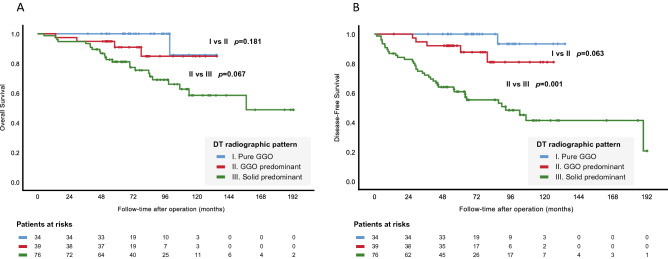
Overall and disease-free survival between different radiologic appearances (**A**, **B**) of the dominate tumor.

**Figure 4:**
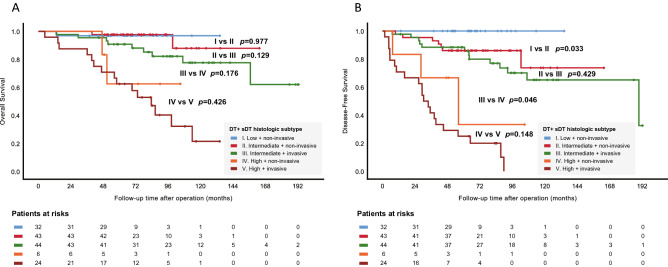
Overall (**A**) and disease-free (**B**) survival between different histologic subtyping combinations.

High-grade DT was correlated with inferior OS and DFS (*p* < 0.001), as shown in Fig. 2A,B. Similarly, DT showing solid-dominant radiologic appearance had inferior DFS (*p* = 0.001) but marginally inferior OS (*p* = 0.067), as shown in Fig. 3A,B. In further analysis taking DT and sDT together, sDT with either non-invasive or invasive histology, regardless of its corresponding DT subtyping, did not influence OS or DFS (Fig. 4A,B). In Fig. 4B, the patients with intermediate-grade DT and non-invasive sDT had significantly poorer DFS than the low-grade DT group of patients (*p* = 0.033), and the patients with intermediate-grade DT and invasive sDT still had significantly better DFS compared with those with high-grade DT and non-invasive sDT. (*p* = 0.046).

Table 3 presents the results of Cox proportional hazard model for DFS in 149 patients. Multivariate analysis revealed high-grade DT as the single-most important predictor of DFS (hazard ratio [HR] 5.324; 95% confidence interval [CI] 2.570–11.462, *p* < 0.001). Age, maximum tumor dimension, solid-dominant radiologic appearance, pleural invasion, poor histology differentiation, angiolymphatic invasion, bilateral tumor distribution, and sDT invasive subtype were all not predictors of DFS. Table 4 depicts the results of Cox proportional hazard model for OS. High-grade DT (HR 3.287; 95% CI 1.323–8.168, *p* = 0.010) and age ≥ 65 (HR 2.823; 95% CI 1.187–6.713, *p* = 0.019) were the most significant predictors of OS.

**Table 3:** Cox proportional hazard model for disease-free survival in 149 patients.

Variable	Univariate			Multivariate		
	HR	95% CI	p value	HR	95% CI	p value
Age (≥ 65 years old)	2.388	1.317–4.329	0.004	1.403	0.736–2.674	0.304
Gender (male)	1.098	0.593–2.033	0.765			
Smoking history	1.592	0.850–2.979	0.146			
Preoperative CEA level (> 6.0 ng/mL)	1.428	0.662–3.083	0.364			
Maximum tumor dimension (> 30 mm)*	2.136	1.139–4.005	0.018	0.973	0.500–1.893	0.936
Radiologic appearance (solid-dominant)*	7.015	2.957–16.644	< 0.001	2.371	0.892–6.307	0.084
Pleural invasion (PL1, PL2, PL3)*	4.226	2.024–8.822	< 0.001	1.648	0.727–3.740	0.232
Histology differentiation (poor)*	3.827	2.071–7.071	< 0.001	1.248	0.607–2.564	0.547
Angiolymphatic invasion*	3.309	1.793–6.107	< 0.001	1.209	0.571–2.438	0.655
Subtyping predominate (high-grade)*	10.066	5.369–18.871	< 0.001	5.324	2.570–11.462	< 0.001
Tumor distribution (bilateral)	0.982	0.495–1.950	0.959			
Tumor located in the same lobe	0.730	0.367–1.450	0.369			
Adjuvant chemotherapy	2.566	1.399–4.706	0.002	0.757	0.387–1.484	0.418
Second dominant subtyping (invasive type)**	4.161	2.046–8.473	< 0.001	1.986	0.902–4.374	0.089

Calculated by Cox regression method.

*CEA* Carcinoembryonic antigen.

*Dominant tumor.

**Second dominant tumor with other than lepidic predominant subtype.

**Table 4:** Cox proportional hazard model for overall survival in 149 patients.

Variable	Univariate			Multivariate		
	HR	95% CI	p value	HR	95% CI	p value
Age (≥ 65 years old)	4.756	2.104–10.751	< 0.001	2.823	1.187–6.713	0.019
Gender (male)	1.667	0.801–3.470	0.172			
Smoking history	2.646	1.273–5.501	0.009	1.719	0.745–3.965	0.204
Preoperative CEA level (> 6.0 ng/mL)	1.632	0.661–4.029	0.288			
Maximum tumor dimension (> 30 mm)*	3.066	1.472–6.384	0.003	1.959	0.911–4.213	0.085
Radiologic appearance (solid)*	4.129	1.566–10.888	0.004	0.724	0.226–2.325	0.588
Pleural invasion (PL1, PL2, PL3)*	3.254	1.374–7.706	0.007	1.888	0.687–5.190	0.218
Histology differentiation (poor)*	3.784	1.781–8.041	0.001	1.410	0.531–3.741	0.490
Angiolymphatic invasion*	2.633	1.232–5.629	0.013	1.041	0.422–2.571	0.930
Subtyping predominate (high-grade)*	7.329	3.426–15.676	< 0.001	3.287	1.323–8.168	0.010
Tumor distribution (bilateral)	0.766	0.311–1.885	0.562			
Tumor located in the same lobe	0.855	0.378–1.934	0.706			
Adjuvant chemotherapy	1.632	0.661–4.029	0.288			
Second dominant subtyping (invasive type)**	4.514	1.706–11.949	0.002	1.879	0.654–5.396	0.241

Calculated by Cox regression method.

*CEA* Carcinoembryonic antigen.

*Dominant tumor.

**Second dominant tumor with other than lepidic predominant subtype.

## Discussion

In this investigation of patients with SMADCs undergoing surgical resection, patients were divided into groups according to radiologic CTR pattern, histologic subtype pattern, and combined histologic pattern. As far as we know, this is the first study to combine dominate lung adenocarcinoma with second dominate adenocarcinoma concurrently to evaluate histologic subtype, rather than radiologic aspect alone. The results of the present study showed that survival outcomes, both DFS and OS, depended definitively on the predominant subtype of histology for grading of DT. Neither could sDT interfere with the patient outcomes. That means, for SMADC patients, further surveillance strategy or adjuvant therapy could be recommended solely based on the histologic grading of DT.

The classification approach based on histologic subtypes can clearly stratify recurrent node-negative lung adenocarcinoma. Additionally, our previous study has demonstrated no significant differences in OS among patients with stage-matched solitary primary lung cancer without mediastinal lymph node involvement^4^. The present study further demonstrated postoperative outcomes in accordance with histologic subtypes of dominate lung adenocarcinoma, rather than tumor size alone. Accordingly, we recommend that rigorous histologic determination and adequate oncologic anatomic resection for DT, as well as sublobar resection for sDT regardless of its histologic pattern, should be taken into account of surgical planning for node-negative SMADCs.

The present study found that smoking history was closely associated with the predominant grading of lung adenocarcinoma, as observed not only in DT but sDT. Similar findings were demonstrated in other studies, and were proposed as a mechanism to predict poor outcome of adenocarcinoma^12,13^. It is therefore crucial for conducting further studies to address the role of smoking in outcomes of adenocarcinoma, especially when the incidence of non-smokers in adenocarcinoma of lung is relatively higher in East Asia.

For accurate estimate of prognosis, the present study excluded IPMs from the analysis of SMADC outcomes, although it is clinically difficult to differentiate SMPLC from stage IV IPMs. For multi-focal lung tumors classified into GGO or pure solid groups, Hattori et al. demonstrated that the pure solid + pure solid type, compared with the other groups with GGOs, showed a clinicopathologically invasive nature^10^. Nearly 25% of the pure solid groups were deemed to have IPM on pathologic analysis rather than SMPLC in other groups with GGOs. Accordingly, when both suspected malignant lesions presented as solid-dominant or pure solid nodules showing no spiculation or air-bronchogram on CT, the likelihood of IPM was high^14,15^. In addition, discordance of driver mutations between SMPLCs in individual patients deserved as favorable prognosis as in patients with only independent primary tumors, which supports different treatment strategies from those for metastatic disease^11^.

Previous studies evaluated DT and sDT mainly based on radiologic features. The presence of GGO components in multiple pulmonary sites indicate a synchronous primary adenocarcinoma^16^. The best survival could be expected in patients with at least one tumor with a GGO component at clinical N0 stage^5^. Gu et al.’s study strongly supported that clinical N0 DT (pathologically adenocarcinoma) with limited, multifocal and in situ adenocarcinomas might enjoy prolonged survival with general anatomic resection of the DT and wedge resection of accessible GGOs^8^. For patients in this group, the presence of secondary tumors had no effect on survival, and surgical therapy was considered^17^. Therefore, excellent surgical outcomes in multifoci GGOs raise other possible strategies to manage this group of patients. For example, we do not need to resect all the foci of GGOs (including sDT) even during a concurrent operation. Observation policy instead of sequential excision for the contralateral sDTs should be considered if DT showed low-grade histological subtyping. Additionally, since most of the residual GGOs made no change or grew slowly during follow-up period^11,17^, observation alone without further adjuvant (or target) therapy for residual GGOs should be advocated, even driver mutations were detected.

In the present study, radiographic appearance of solid-dominant tumors with CTR > 0.5 presented poorer OS and DFS, as similarly noted in the Japan Clinical Oncology Group Lung Cancer Surgical Study Group^18^. However, further multiple Cox-regression analysis did not confirm it as the same important predictor of survival as histologic subtyping did. In Kim et al.’s study, CTR was not an independent prognostic factor of surgically treated adenocarcinomas when clinical T factor (from 8th edition AJCC TNM classification system) was included in the survival analysis for adjustment^19^. For assessing tumor characteristics, an optimal method of imaging analysis is crucial but still awaits further study for verification. In other words, radiologic measurements alone cannot precisely predict the invasive status of tumors before patients undergo surgery^20^. Even pre-operative tissue biopsy can only provide additional information about the invasiveness of the subtypes^21^. Furthermore, final results of several clinical trials evaluating the efficacy of limited surgery are still pending^22^. Solid-dominant tumors on CT had higher potential for malignancy, which might not be suitable for limit resection^16^. The analysis of sDT lung adenocarcinoma in the present study did not weaken the power of our findings, but optimal treatment methods are still controversial.

### Limitations

The present study has several limitations. First, this is a retrospective study in a single institution, which limits inferences of causality and generalization to other populations. Patients had all undergone surgical intervention and were diagnosed based on histopathology of lung adenocarcinoma; therefore, direct comparison with radiologic or other diagnostic methods was not performed. Second, patients included in the present study had at least 2 pulmonary tumors resected, and many patients with the potential for residual GGOs did not undergo surgery. Although these situations seemed not to impact on outcome analysis according to experiences from previous studies, the possibilities of bias cannot be ruled out. Finally, the present study investigated the IASLC/ATS/RES histological classification, rather than radiologic patterns. Surgical planning and decision making is still difficult by evaluating the component of GGO and CT scanning technology. The correlation between radiologic findings and pathologic results is still controversial.

## Conclusion

In conclusion, micropapillary or solid predominate subtypes of DT are the most important predictors of surgical outcomes in patients with SMADCs. Neither solid-dominate radiologic appearance nor sDT subtype is a prognostic factor.

## Supplementary Information

Supplementary Legend.

Supplementary Figure 1.

## References

[1] Jung EJ (2011). Treatment outcomes for patients with synchronous multiple primary non-small cell lung cancer. Lung Cancer.

[2] Martini N, Melamed MR (1975). Multiple primary lung cancers. J. Thorac. Cardiovasc. Surg..

[3] Travis WD (2015). The 2015 World Health Organization classification of lung tumors: Impact of genetic, clinical and radiologic advances since the 2004 classification. J. Thorac. Oncol..

[4] Yu YC (2013). Surgical results of synchronous multiple primary lung cancers: Similar to the stage-matched solitary primary lung cancers?. Ann. Thorac. Surg..

[5] Matsunaga T, Suzuki K, Takamochi K, Oh S (2017). New simple radiological criteria proposed for multiple primary lung cancers. Jpn. J. Clin. Oncol..

[6] Liu Y (2016). Genomic heterogeneity of multiple synchronous lung cancer. Nat. Commun..

[7] Ishikawa Y (2014). Surgical treatment for synchronous primary lung adenocarcinomas. Ann. Thorac. Surg..

[8] Gu B (2013). A dominant adenocarcinoma with multifocal ground glass lesions does not behave as advanced disease. Ann. Thorac. Surg..

[9] Hung JJ (2014). Predictive value of the international association for the study of lung cancer/American Thoracic Society/European Respiratory Society classification of lung adenocarcinoma in tumor recurrence and patient survival. J. Clin. Oncol..

[10] Hattori A, Takamochi K, Oh S, Suzuki K (2020). Prognostic classification of multiple primary lung cancers based on a ground-glass opacity component. Ann. Thorac. Surg..

[11] Chen K (2018). Favorable prognosis and high discrepancy of genetic features in surgical patients with multiple primary lung cancers. J. Thorac. Cardiovasc. Surg..

[12] Yi JH (2019). Prognostic significance of cigarette smoking in association with histologic subtypes of resected lung adenocarcinoma. Korean J. Thorac. Cardiovasc. Surg..

[13] Sakao Y (2008). The impact of cigarette smoking on prognosis in small adenocarcinomas of the lung: The association between histologic subtype and smoking status. J. Thorac. Oncol..

[14] Suh YJ (2020). A novel algorithm to differentiate between multiple primary lung cancers and intrapulmonary metastasis in multiple lung cancers with multiple pulmonary sites of involvement. J. Thorac. Oncol..

[15] Yu YC, Huang CS, Huang BS (2018). Separate or intrapulmonary metastasis?. J. Thorac. Dis..

[16] Shimada Y (2015). Survival of a surgical series of lung cancer patients with synchronous multiple ground-glass opacities, and the management of their residual lesions. Lung Cancer.

[17] Stiles BM (2015). Characteristics and outcomes of secondary nodules identified on initial computed tomography scan for patients undergoing resection for primary non-small cell lung cancer. J. Thorac. Cardiovasc. Surg..

[18] Ito H (2020). Long-term survival outcome after lobectomy in patients with clinical T1 N0 lung cancer. J. Thorac. Cardiovasc. Surg..

[19] Kim H, Goo JM, Kim YT, Park CM (2019). Consolidation-to-tumor ratio and tumor disappearance ratio are not independent prognostic factors for the patients with resected lung adenocarcinomas. Lung Cancer.

[20] Ye T (2018). Predictors of pathologic tumor invasion and prognosis for ground glass opacity featured lung adenocarcinoma. Ann. Thorac. Surg..

[21] Tsai PC (2020). CT-guided core biopsy for peripheral sub-solid pulmonary nodules to predict predominant histological and aggressive subtypes of lung adenocarcinoma. Ann. Surg. Oncol..

[22] Kobayashi Y, Ambrogio C, Mitsudomi T (2018). Ground-glass nodules of the lung in never-smokers and smokers: Clinical and genetic insights. Transl. Lung Cancer Res..

