# Supplementary material for: Prognostic histologic subtyping of dominant tumor in resected synchronous multiple adenocarcinomas of lung
Source: Sci Rep. 2021 May 5;11:9539. doi: 10.1038/s41598-021-88193-9 (PMC8100294; doi:10.1038/s41598-021-88193-9)
Supplement: Supplementary file 1 — Supplementary Legend. [file 41598_2021_88193_MOESM1_ESM.docx]

Supplementary Figure 1: The histologic relevance between DTs and sDTs
