# Supplementary figures and images for: Prognostic histologic subtyping of dominant tumor in resected synchronous multiple adenocarcinomas of lung
Source: Sci Rep. 2021 May 5;11:9539. doi: 10.1038/s41598-021-88193-9 (PMC8100294; doi:10.1038/s41598-021-88193-9)

Intermediate grade of DT

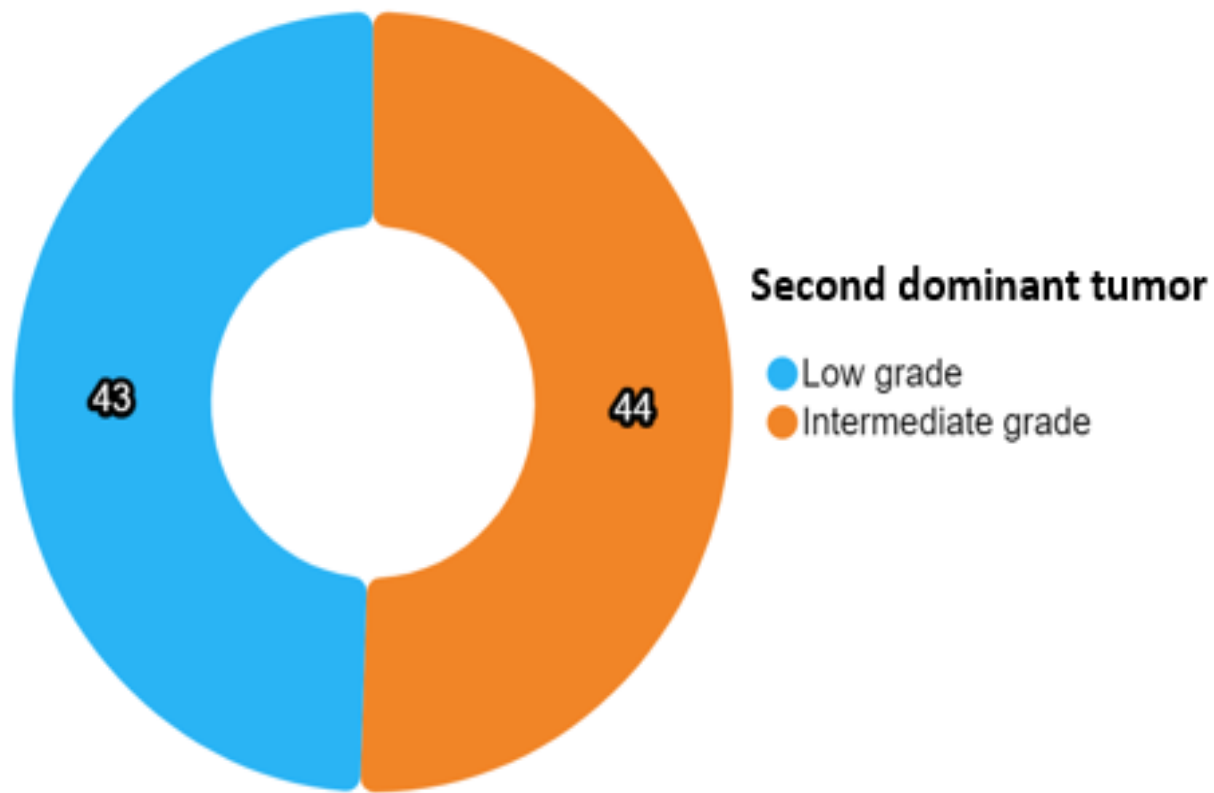

High grade of DT

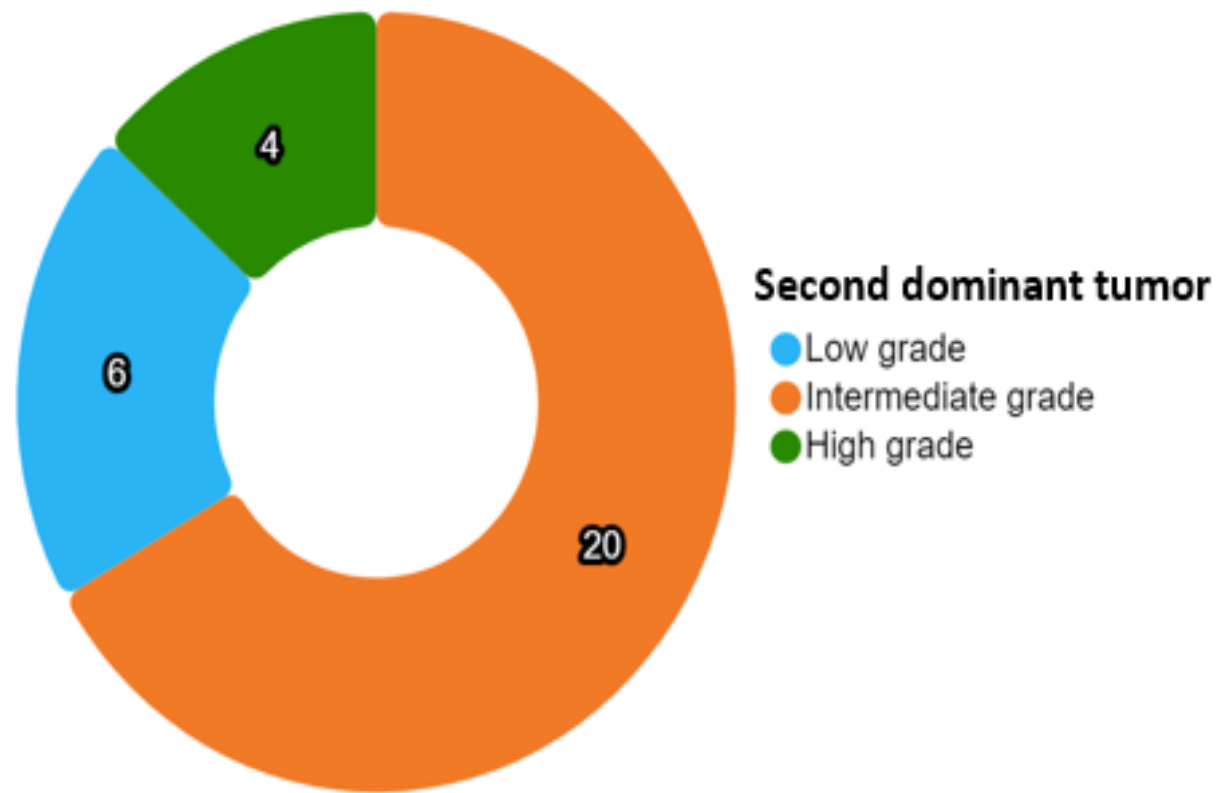

Supplement: Supplementary file 2 — Supplementary Figure 1. [file 41598_2021_88193_MOESM2_ESM.pdf]
